# Fibroblast growth factor receptor 1 gene amplification is associated with poor survival in patients with resected esophageal squamous cell carcinoma

**DOI:** 10.18632/oncotarget.2944

**Published:** 2014-12-10

**Authors:** Hyo Song Kim, Seung Eun Lee, Yoon Sung Bae, Dae Joon Kim, Chang-Geol Lee, Jin Hur, Hyunsoo Chung, Jun Chul Park, Da Hyun Jung, Sung Kwan Shin, Sang Kil Lee, Yong Chan Lee, Hye Ryun Kim, Yong Wha Moon, Joo Hang Kim, Young Mog Shim, Susan S. Jewell, Hyunki Kim, Yoon-La Choi, Byoung Chul Cho

**Affiliations:** ^1^ Division of Medical Oncology, Department of Internal Medicine, Yonsei University College of Medicine, Seoul, Korea; ^2^ Departments of Pathology, Samsung Medical Center, Sungkyunkwan University School of Medicine, Seoul, Korea; ^3^ Department of Pathology, Yonsei University College of Medicine, Seoul, Korea; ^4^ Department of Thoracic and Cardiovascular Surgery, Yonsei University College of Medicine, Seoul, Korea; ^5^ Department of Radiation Oncology, Yonsei University College of Medicine, Seoul, Republic of Korea; ^6^ Department of Radiology, Yonsei University College of Medicine, Seoul, Republic of Korea; ^7^ Division of Gastroenterology, Department of Internal Medicine, Yonsei University College of Medicine, Seoul, Korea; ^8^ Department of Thoracic Surgery, Samsung Medical Center, Sungkyunkwan University School of Medicine, Seoul, Korea; ^9^ Abbott Molecular Laboratories, Des Plaines, IL

**Keywords:** Fibroblast growth factor receptor 1, esophageal squamous cell carcinoma, gene amplification, fluorescent in situ hybridization, prognostic factor

## Abstract

To investigate the frequency and the prognostic impact of *fibroblast growth factor receptor 1* (*FGFR1*) gene amplification in 526 curatively resected esophageal squamous cell carcinoma (ESCC). Using fluorescent in situ hybridization, high amplification was defined by an *FGFR1*/centromer 8 ratio is ≥ 2.0, or average number of *FGFR1* signals/tumor cell nucleus ≥ 6.0, or percentage of tumor cells containing ≥ 15 *FGFR1* signals or large cluster in ≥ 10%. Low amplification was defined by ≥ 5 *FGFR1* signals in ≥ 50%. *FGFR2* and *FGFR3* mutations were assessed by direct sequencing in 388 cases and no mutation was detected. High and low amplification were detected in 8.6% and 1.1%, respectively. High *FGFR1* amplification had significantly shorter disease-free survival (34.0 *vs* 158.5 months *P*=0.019) and overall survival (52.2 *vs* not reached *P*=0.022) than low/no amplification group. After adjusting for sex, smoking, stage, histology, and adjuvant treatment, high *FGFR1* amplification had a greater risk of recurrence (adjusted hazard ratio [AHR], 1.6; *P*=0.029) and death (AHR, 1.53; *P*=0.050). High amplification was significantly higher in current smokers than former and never-smokers (*P*_trend_<0.001) and increased proportional to smoking dosage. High *FGFR1* amplification is a frequent oncogenic alteration and an independent poor prognostic factor in resected ESCC.

## INTRODUCTION

Esophageal cancer (EC) is the sixth most common cause of cancer death worldwide [1]. Despite the improvement of surgical technique and medical treatment, prognosis remains poor, with 5-year survival rate of less than 40%.[2] EC consists of two major histologic subtypes; esophageal squamous cell carcinoma (ESCC) and adenocarcinoma (EAC). Both histologic subtypes have different risk factors and epidemiology. ESCC continues to be the major subtype of EC in Asia, and the main risk factors include smoking and alcohol abuse.[3] In contrast, EAC predominately affects the Caucasian, and is closely associated with gastroesophageal reflux disease, Barrett's esophagus, and obesity. [4-7] Compared with ESCC, EAC shows younger age onset, male predominance and frequent occurrence in distal third of the esophagus.[8] Given widely different epidemiological and clinical features, EAC and ESCC may represent distinct disease entities which may benefit from different therapeutic approaches.

Amplification and overexpression of human epidermal growth factor receptor 2 (HER2; also known as ERBB2) was more frequent in EAC than ESCC and there was a strong concordance of *HER2* status in primary and metastatic cancer.[9] Therefore, various clinical trials evaluating the efficacy of HER2 targeting agents were reported for the patients with advanced esophageal and esophagogastric junction (EGJ) adenocarcinoma.^8-10^ In the phase II trials for HER2 amplified EGJ adenocarcinoma, lapatinib, a dual tyrosine kinase inhibitor of ERBB1 and HER2 demonstrated modest response.[10, 11] The therapeutic implication of HER2 protein overexpression or gene amplification has been demonstrated in the Trastuzumab for Gastric Cancer (ToGA) trial [12]. In this trial, addition of trastuzumab (anti-HER2 antibody) to standard chemotherapy significantly improved response rate, progression-free survival and overall survival compared with chemotherapy alone in HER2-positive advanced gastric and EGJ adenocarcinoma. Based on the results of the ToGA trial, HER2 testing is routinely recommended for all patients with metastatic gastric or EGJ adenocarcinoma, and trastuzumab should be considered for HER2-positive cases.

Despite a rapid and enthusiastic development of targeted therapies in gastric or EGJ adenocarcinoma, no therapeutically tractable target has been identified for ESCC. Recently, comparative genomic analysis showed different DNA copy number alterations between EAC and ESCC.[13, 14] Among those, high copy number gains of cancer-associated genes, such as *SOX2, PIK3CA, CCND1*, and *FGFR1*, were more frequently observed in ESCC than in EAC, suggesting that genomic gain of these oncogenes may be therapeutic targets for ESCC.

Fibroblast growth factor receptor 1 (FGFR1) is a member of family of receptor tyrosine kinases (FGFR1-4), and its activation by amplification, mutation, or translocation leads to tumor cell proliferation and survival in many cancers.[15, 16] Potentially actionable FGFR rearrangements were identified in diverse solid tumors including lung cancer, oral cancer, and breast cancer. [17-19] *FGFR1* amplification has been frequently reported in lung squamous cell carcinoma (SqCC), small cell lung cancer, and SqCC of head and neck (SCCHN), for which smoking is a clear and dominant risk factor.[15, 16, 20-28] Overall, the frequency of *FGFR1* amplification was reported to be 5.6%-24.8% in lung SqCC and 15%-17.4% in SCCHN, suggesting that this genetic alteration mainly target squamous cell histology.[16, 20-25, 27, 28] Furthermore, *FGFR1* amplification has been associated with poor prognosis or unfavorable clinicopathologic parameters in lung SqCC and SCCHN. Because ESCC has risk factors in common with lung SqCC and SCCHN, we hypothesized that *FGFR1* amplifications is associated with pathogenesis and poor prognosis in ESCC.

In this study, we sought to determine the frequency, prognostic impact and association with smoking dosage of *FGFR1* amplification in surgically resected ESCC. Furthermore, we also evaluated the frequency of *FGFR2* and *FGFR3* mutations in ESCC.

## RESULTS

### Patient Characteristics

A total of 526 Korean patients who underwent curative esophagectomy were analyzed. The clinical characteristics of the enrolled patients are presented in Table 1. There were 489 male and 37 female with a median age of 66 years (range 35-98). Median tumor size was 3 cm and approximately half of tumors had pT3 or pN0. Pathologic stages were I in 22.4%, II in 42.8%, and III in 34.8%. Two thirds (60.3%) were located in the lower esophagus, and more than half of patients had moderately differentiated SqCCs. The majority of patients were current (39.0%) or former smokers (38.6%), and median smoking dosage was 30.0 pack-years (range 0-150). Adjuvant treatment was given in 140 patients (26.6%), and 87 of those (62.1%) were treated by concurrent chemoradiotherapy. The proportions of adjuvant therapy according to stage were 5.9% in stage I, 21.8% in stage II, and 45.9% in stage III.

**Table 1:** Patient characteristics according to FGFR1 amplification

Characteristics	All patients		High amplification*		Low amplification*		No amplification		

	No.	%	No.	%	No.	%	No.	%	P^†^
No of patients	526	100	45	8.6	6	1.1	475	90.3	
Age, years									0.697
Median (range)	66 (35-98)		67 (52-77)		68 (65-77)		66 (35-98)		
Sex									0.608
Male	489	93.0	43	95.6	6	100	440	92.6	
Female	37	7.0	2	4.4	0	0	35	7.4	
Tumor size, cm									0.310
Median, range	3.0 (0.2-10.5)		3.0 (2.0-9.0)		2.5 (2.0-4.5)		3.0 (0.2-10.5)		
pT stage^‡^									0.618
T1	167	31.7	12	26.7	2	33.3	153	32.2	
T2	106	20.2	12	26.7	1	16.7	93	19.6	
T3	238	45.2	18	40.0	3	50.0	217	45.7	
T4	15	2.9	3	6.7	0	0	12	2.5	
pN stage^‡^									0.619
N0	259	49.2	22	48.9	3	50.0	234	49.3	
N1	234	44.5	20	44.4	2	33.3	212	44.6	
N2	22	4.2	3	6.7	1	16.7	18	3.8	
N3	11	2.1	0	0	0	0	11	2.3	
pTMN stage^‡^									0.822
I	118	22.4	8	17.8	1	16.7	109	22.9	
II	225	42.8	19	42.2	2	33.3	204	42.9	
III	183	34.8	18	40.0	3	50.0	162	34.1	
Location									0.158
Upper	66	12.5	5	11.1	0	0	61	12.8	
Middle	143	27.2	19	42.2	2	33.3	122	25.7	
Lower	317	60.3	21	46.7	4	66.7	292	61.5	
Tumor grade									0.424
Well	128	24.3	12	26.7	2	33.3	114	24.0	
Moderate	314	59.7	29	64.4	4	66.7	281	59.2	
Poorly	84	16.0	4	8.9	0	0	80	16.8	
Smoking status^¶^									<0.001
Never-smoker	118	22.4	1	2.2	0	0	117	24.6	
Former smoker	203	38.6	5	11.1	2	33.3	196	41.3	
Current smoker	205	39.0	39	86.7	4	66.7	162	34.1	
Smoking dosage, pack-years									
Median	30		39		35		30		0.002
Range	0-150		0-99		13-60		0-150		
Adjuvant therapy									0.870
Yes	140	26.6	13	28.9	2	33.3	125	26.3	
No	386	73.4	32	71.1	4	66.7	350	73.7	
*FGFR1* FISH^§^									
Number (median, range)	2.2 (0-15.5)		6.4 (4.1-15.5)		5.1 (5.0-5.6)		2.2 (0-5.7)		<0.001
Ratio (mean, range)	1.5 (0-7.8)		2.9 (1.1-7.8)		1.5(1.0-1.9)		1.3 (0-1.6)		0.003

Abbreviations:

* High *FGFR1* amplification was defined as if one of the following criteria is fulfilled: (1) *FGFR1*/CEN8 ratio is ≥ 2.0, (2) average number of *FGFR1* signal per nucleus ≥ 6.0, and (3) percentage of tumor cells containing ≥ 15 *FGFR1* signals or large clusters in ≥ 10% cells. Low *FGFR1* amplification was defined when the percentage of tumor cells containing ≥ 5 *FGFR1* signals is ≥ 50 %.

† χ^2^ test, Fisher's exact test, or Mann-Whitney U test.

‡ Pathologic stage at the time of surgical resection was determined according to the American Joint Committee on Cancer (seventh edition) guidelines.

¶ Never-smokers; a lifetime smoking dose of fewer than 100 cigarettes; former smokers, those who have stopped smoking for more than 1 year; current smokers, those who currently smoke or have quit for less than 1 year.

§ *FGFR1* numbers are average numbers of *FGFR1* signals per nucleus, and ratios are *FGFR1*/CEN8 ratios.

### *FGFR1* amplification status and clinicopathologic features

**Figure 1:**
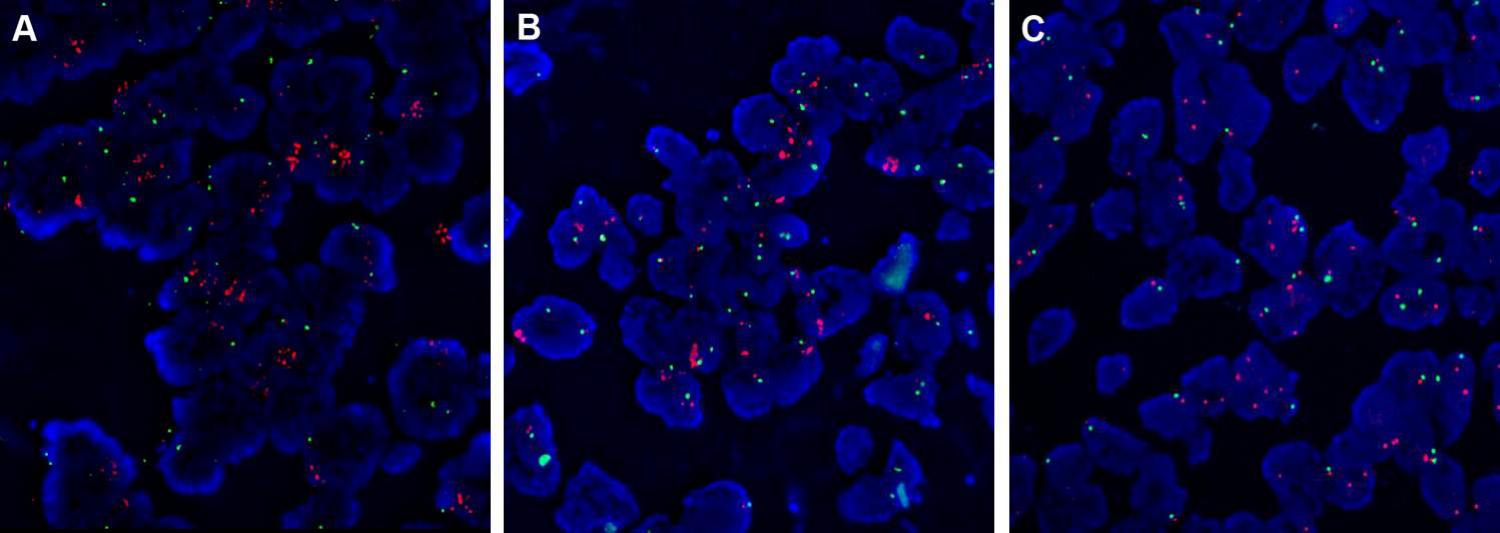
Fibroblast growth factor receptor 1 (*FGFR1*) amplification assessed by fluorescent in situ hybridization (A) High *FGFR1* amplification; (B) Low *FGFR1* amplification; (C) No amplification.

Among 526 patients, 45 (8.6%) were high *FGFR1* amplification, 6 (1.1%) were low *FGFR1* amplification, and 475 (90.3%) displayed no amplification (Table 1; Figure 1). The median *FGFR1* gene copy number per nucleus and the mean *FGFR1*/CEN8 ratio in all patients were 2.2 (range, 0 to 15.5 copies per nucleus) and 1.5 (range, 0 to 7.8). The median *FGFR1* gene copy number was 6.4 (range, 4.1 to 15.5) in high amplification, 5.1 (range, 5.0 to 5.6) in low amplification, and 2.2 (range 0 to 5.7) in no amplification group. The mean *FGFR1*/CEN8 ratio was 2.9 (range 1.1 to 7.8), 1.5 (range, 1.0 to 1.9), and 1.3 (range, 0 to 1.6) in high, low and no amplification group, respectively. Of 45 high *FGFR1* amplified tumors, 12 cases (26.7%) only satisfied the criterion of an *FGFR1*:CEN8 ratio of ≥ 2.0, 7 cases (15.6%) only satisfied the criterion of an average number of *FGFR1* signal per nucleus *≥*6.0, and 4 cases (8.9%) only satisfied the criterion of percentage of tumor cells containing ≥ 15 *FGFR1* signals or large clusters in ≥ 10% cells, respectively. 22 cases (48.9%) satisfied all three criteria for *FGFR1* amplification simultaneously.

There was no significant difference among three *FGFR* groups regarding age, sex, tumor size, pathologic stage, location, histologic grade, and adjuvant therapy (Table 1). However, the incidence of high *FGFR1* amplification was significantly higher in smokers than in never-smokers (P<0.001). Only 1 (2.2%) of 45 patients with high-level amplification was never smoker. The median smoking dosage was significantly higher for high amplification group than that for low or no amplification group(39 *vs* 35 *vs* 30 pack-years, *P*=0.002). Based on the pattern of high amplification (group fulfilling 3 criteria *vs.* 1 or 2 criteria), there was no significant difference in terms of age, sex, location, and pathologic stage.

### Survival Outcomes According to *FGFR1* Amplification

With a median follow-up time of 55.4 months, the 5 year DFS and OS rates for all patients were 52.8% and 59.8%, respectively. The 5-year DFS rate according to pTNM stages were 78.4% in stage I, 55.9% in stage II, and 33.2% in stage III patients. The 5-year OS rate according to pTNM stages were 86.8% for stage I, 65.3% for stage II, and 37.1% for stage III.

**Figure 2:**
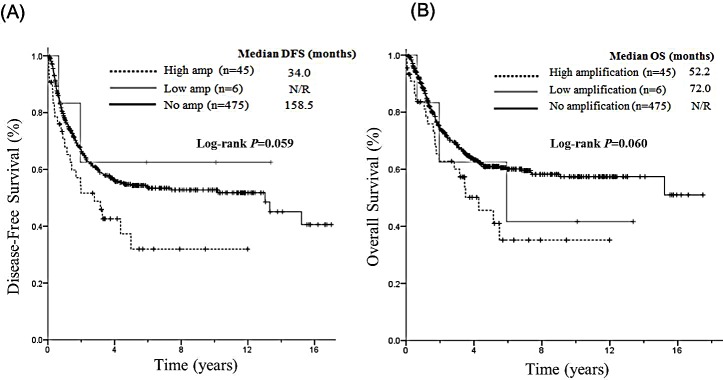
Survival analysis on the basis of *FGFR1* amplification (high, low, and no amplification) (A) Median DFS was 34.0 months in the high *FGFR1* amplification group, not reached in low amplification group, and 158.5 months in the no amplification group. (B) The median OS was 52.2 months in the high *FGFR1* amplification group, and 72.0 months in the low amplification group, and not reached in the no amplification group.

The median DFS for each of the three *FGFR* groups was 34.0 months in the high *FGFR1* amplification, not reached (NR) in low amplification group, and 158.5 months in the no amplification group (Figure 2A). By using pair-wise comparison, patients with high *FGFR1* amplification showed a significantly shorter DFS than those with no amplification (34.0 *vs* 158.5 months in no amplification, *P=*0.020). The median DFS of high amplification and low amplification were not significantly different (34.0 *vs* NR in low amplification, *P*=0.272), probably due to small sample size in the low amplification group. The median DFS was similar between low amplification and no amplification group in pair-wise comparison (*P*=0.639). The median OS for each of the three *FGFR* groups was 52.2 months in the high *FGFR1* amplification, 72.0 months in the low amplification, and not reached in the no amplification (Figure 2B). By using pair-wise comparison, patients with high *FGFR1* amplification showed a significantly shorter OS than those with no amplification (52.2 *vs* NR in no amplification, *P*=0.021). The OS times of high amplification and low amplification were not significantly different (52.2 *vs* 72.0 in low amplification, *P*=0.637), probably due to small sample size in the low amplification group. The median OS was similar between low amplification and no amplification group in pair-wise comparison (*P*=0.517).

**Figure 3:**
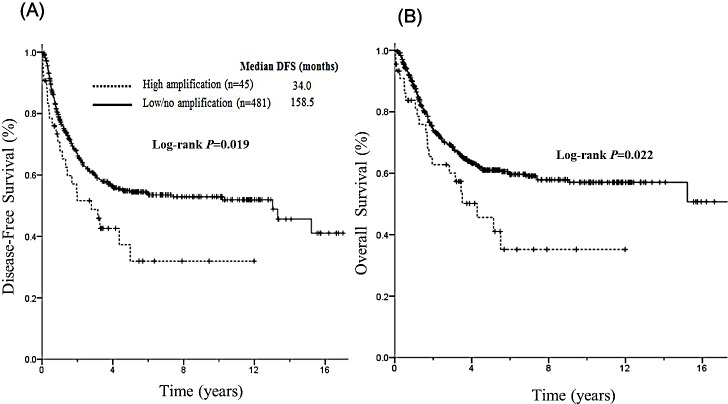
Prognostic impact of high *FGFR1* amplification and low/no amplification on DFS and OS (A) The median DFS of the high *FGFR1* amplification group was significantly shorter compared with low/no *FGFR1* amplification group (*P*=0.019) (B) The median OS of high *FGFR1* amplification group demonstrated significantly shorter than low/no *FGFR1* amplification group (*P*=0.022).

Because the DFS of patients with low and no amplification were similar but different from that of patients with high amplification, we categorized total patients into high amplification group and low/no amplification group and assessed the survival outcomes of these two groups. The median DFS of the high *FGFR1* amplification group was significantly shorter compared with that of the low/no *FGFR1* amplification group (34.0 *vs* 158.5 months *P*=0.019, Figure 3A). In addition, high *FGFR1* amplification group demonstrated significantly shorter OS than low/no *FGFR1* amplification group (52.2 vs NR, *P*=0.022, Figure 3B). In Cox proportional hazard model adjusted for sex, smoking history, pathologic stage, adjuvant treatment, and histologic grade, high *FGFR1* amplification was significantly associated with a shorter DFS (AHR 1.61; 95% CI, 1.05-2.46; *P*=0.029, Table 2). There was a strong trend toward worse OS for high *FGFR1* amplification compare to low/no *FGFR1* amplification group in multivariate analysis (HR 1.53; 95% CI, 0.99-2.34, *P*=0.050). There was no significant difference in DFS and OS for sex, smoking status, and adjuvant treatment in multivariate analysis. There was no significant difference in DFS and OS between group fulfilling 3 criteria and group fulfilling 1 or 2 criteria.

**Table 2:** Survival outcome in multivariate analysis

		DFS			OS		
Variable	Category	HR	95% CI	P	HR	95% CI	P
Sex	Male vs female (ref)	0.91	0. 53-1.55	0.724	0.90	0.53-1.54	0.703
Smoking status	Current smoker *vs* never/former-smoker (ref)	1.08	0.82-1.43	0.595	1.02	0.77-1.35	0.898
Pathologic stage*	III vs II/I (ref)	2.56	1.97-3.34	<0.001	2.53	1.94-3.29	<0.001
Histology	Poor vs well/moderate(ref)	1.61	1.16-2.22	0.004	1.61	1.17-2.23	0.004
Adjuvant treatment	Yes vs no (ref)	1.11	0.82-1.50	0.497	1.05	0.78-1.43	0.731
*FGFR1* amplification	High vs Low /no (ref)	1.61	1.05-2.46	0.029	1.53	0.99-2.34	0.050

Abbreviations: DFS, disease-free survival; OS, overall survival; ref, reference; amp, amplification;

* Clinical stage at the time of initial diagnosis was determined according to the American Joint Committee on Cancer (seventh edition) guidelines.

### *FGFR2* and *FGFR3* mutations

*FGFR2* and *FGFR3* mutations were assessed by direct sequencing in 388 cases with FFPE tissues available and no *FGFR2* or *FGFR3* mutation was detected.

### Association between *FGFR1* amplification and smoking

**Figure 4:**
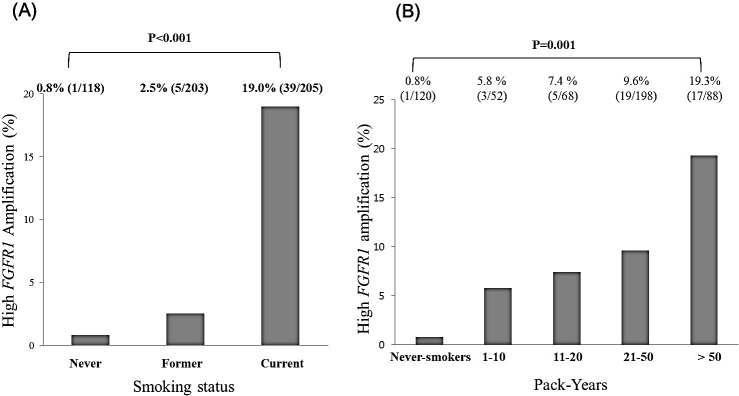
Association of smoking status and high *FGFR1* amplification (A) Proportions of high *FGFR1* amplification according to never-, former, and current smokers (B) Incidence of high *FGFR1* amplification according to smoking dosage.

As shown in Figure 4A, the incidence of high-level *FGFR1* amplification was significantly higher in current smokers than former or never-smokers (*P*_trend_ <0.001). The incidences of high-level *FGFR1* amplification in current, former and never-smokers were 19.0%, 2.5%, and 0.8%, respectively. With increment of total cigarette smoking dosage, the incidence of high-level *FGFR1* amplification was significantly increased (Figure 4B, *P*_trend_ = 0.001). High-level *FGFR1* amplification were 0.8% for never-smokers, 5.8% in patients with 1-10 pack-years, 7.4% with 11-20 pack-years, 9.6% with 21-50 pack-years, and 19.3% with more than 50 pack-years.

## DISCUSSION

In this study, we investigated the frequency and the prognostic impact of *FGFR1* amplification in resected ESCC. To our knowledge, this is the first report on the prognostic impact of *FGFR1* amplification in the largest-ever cohort of resected ESCC patients from East Asian. Our study demonstrated high *FGFR1* amplification is a common genetic alteration (8.6%) and an independent negative prognostic factor in resected ESCC. Interestingly, it was associated with cigarette smoking in a dose-dependent manner.

The frequency of *FGFR1* amplification has been reported to range from 6% to 9.4%. In the DNA array hybridization study,[29] 2 out of 32 ESCC cases (6%) demonstrated amplified 8p11 locus which containing *FGFR1* gene. A comparative genomic study revealed many focal DNA amplifications or losses such as *SOX2, PIK3CA, CCND1, FGFR1, MYC, GATA4*, and *GATA6* in ESCC and EAC.[13] Of note, *FGFR1* was amplified in 21% of ESCC samples compare with 8% in EAC. Recently, *FGFR1* amplification was reported to be 9.4% in the cohort with Western Europe.[30] The frequency of high *FGFR1* amplification (8.6%), as determined by FISH analysis in our study, was comparable to those reported in previous studies.[13, 29, 30] Given the relatively high frequency of *FGFR1* amplification, it may represent an attractive therapeutic target for ESCC.

Given the histological similarity and common risk factors, it is not surprising that *FGFR1* amplification has been reported in upper aerodigestive tract cancers such as lung SqCC, SCCHN, and small cell lung cancer.[16, 20-28] However, the prognostic significance of *FGFR1* amplification in these cancers has shown controversial results. In resected lung SqCC, Kim et al[16] reported *FGFR1* amplification as negative prognostic factor, whereas Heist et al [24] observed no significant difference in OS. In SCCHN, *FGFR1* amplification was significantly associated with poor prognostic factors such as higher T stage, lymphovascular invasion, and higher numbers of visceral metastases.[22]Tumor heterogeneity, unstandardized FISH criteria for *FGFR1* amplification, varying adjuvant treatment, and small sample size may contribute to the controversial results. In our study, by applying more sophisticated criteria for *FGFR1* amplification, we were able to divide *FGFR1* amplification into high and low amplification. Low *FGFR1* amplification occurred only in 1.1% and survival outcome of low *FGFR1* amplification group was similar to no amplification group. Compared with low/no amplification group, high *FGFR1* amplification was significantly associated with shorter DFS regardless of sex, histology, and adjuvant therapy, implying that *FGFR1* amplification as an independent negative prognostic factor in curatively resected ESCC.

The identification of FGFR alteration in human cancers led to rapid development of selective FGFR inhibitor such as BGJ398 and AZD4547. In phase I study with BGJ398,[31] 1 patient with *FGFR1* amplified lung SqCC with *FGFR1*/CEN8 ratio of 2.6 by FISH analysis had partial response. In another phase I study with FGFR inhibitor AZD4547,[32] 1 partial response was also seen in lung SqCC patient with *FGFR1* amplification (*FGFR1*/CEN8 ratio > 2.8). Various FGFR inhibitors are currently in clinical development for the patients with FGFR aberrations. Therefore, our study strongly suggests the therapeutic potential of FGFR inhibitor for ESCC, and future clinical trials for advanced high *FGFR1* amplified ESCC are strongly warranted.

Unfortunately, FISH criteria for *FGFR1* amplification has not yet been standardized. Indeed, definition of *FGFR1* amplification by FISH technique has been highly variable in the previous studies in lung cancer and breast cancer.[16, 24, 25, 28, 33, 34] Unlike breast cancer, lung SqCC exhibits small-clusters and co-amplifications of *FGFR1* and CEN8.[25] Therefore, *FGFR1* FISH assay needs to differentiate between true amplification and polysomy. In a large cohort study, Schildhause *et al* [25] proposed a more sophisticated *FGFR1* FISH criteria using average gene copy number per nucleus, *FGFR1*/CEN8 ratio, and percentage of gene clusters at the same time. By the addition of *FGFR1*/CEN8 ratio, 8 out of 47 cases (17.0%) were newly classified as high amplification in this study.(25) Similarly, in our study, 12 out of 45 high amplification group (26.7%) might have been misclassified as low/no amplification group if the criterion for *FGFR1/*CEN8 ratio was not included. Considering clinical benefit appeared in patients with high *FGFR1*/CEN8 ratio in clinical trials with FGFR inhibitors,[31, 32] this scoring system might serve as a standardized screening tool to select patients who gain greater benefit from treatment with FGFR inhibitors. Our criteria need further validation in the future clinical trials with FGFR inhibitors in ESCC patients.

Accumulating evidence has shown that *FGFR1* amplification correlated with smoking status in squamous cell biology. *FGFR1* amplification occurs significantly more often in the smokers of the lung SqCC, SCCHN, and small cell lung cancer.[16, 22, 23, 26] The proportion of *FGFR1* amplification among current smokers were reported 15.8% to 28.9% in lung SqCC and 17.7% in SCCHN in a dose dependent manner.[16, 22, 23] In small cell lung cancer cohort, all *FGFR1* amplified cases were current or former smokers.[26] In our study, *FGFR1* amplification was significantly more likely to be smokers, and 44 out of 45 high *FGFR1* amplification cases were current or former smokers. Similar to the findings in lung SqCC and SCCHN, acquisition of *FGFR1* amplification in our study was also significantly increased proportional to smoking dosage. Therefore, *FGFR1* amplification may be an oncogenic driver mutation in tobacco-associated cancers of the aerodigestive tract. An interesting question of the role of *FGFR1* amplification on the smoking-associated carcinogenesis still remained to be solved.

Somatic mutations of *FGFR2* and *FGFR3* were reported in 12% and 30% of endometrial and urothelial cell carcinomas, respectively.[35, 36] Inhibitor–sensitive 5 mutation loci for *FGFR2* and 6 mutation loci for *FGFR3* were noticed in 3% of lung SqCC samples.[37]Here, we show no *FGFR2* and *FGFR3* mutations in our 388 ESCC patients. This may be explained by the limited sensitivity of the Sanger sequencing, tumor heterogeneity or molecular difference of ESCC from lung SqCC.

The main limitation of our study include its retrospective nature and patient selection. Therefore, our findings should be validated in an independent cohort and response data to FGFR-targeted therapies in the future clinical trials.

In conclusion, we demonstrated high *FGFR1* amplification is an independent poor prognostic factor in resected ESCC. Patients with *FGFR1* amplification were significantly more likely to be smokers and the frequency of *FGFR1* amplification was also increased proportional to smoking dosage, suggesting *FGFR1* amplification as an oncogenic aberration induced by smoking carcinogen. Our finding indicates *FGFR1* amplification is a promising therapeutic target in ESCC.

## PATIENTS AND METHODS

### Patients and tissue samples

This study was conducted in a cohort of patients with ESCC who underwent radical esophagectomy at Severance Hospital and Samsung Medical Center, Seoul, Korea, between 2002 and 2010. The criteria used for patient selection included (1) surgically resected SqCC of the thoracic esophagus (R0 resection), (2) availability of primary tumor tissue, (3) no distant metastasis, and (4) no preoperative treatment. The tumor samples of 664 patients were available for examination of *FGFR1* amplification. We excluded 115 cases (17.3%) who received neoadjuvant treatment. Twenty-three patients (3.5%) were excluded because of incomplete survival follow-up and/or smoking data. All diagnosis were reviewed by two experienced pathologist (Y.L.C. and H.K.K) and confirmed by hematoxylin and eosin staining. Three representative cores measuring in 2-mm-diameter for each paraffin-embedded primary tumor were assembled into tissue microarray blocks.

Patients' information was collected by reviewing the medical records for evaluation of clinicopathologic characteristics and survival outcome. Staging was determined using the 7^th^ edition American Joint Committee on Cancer guideline of tumor, node, and metastasis (TNM) classification. Smoking status such as never-smoker, former smoker, and current smoker were defined as previous studies.[38] The study was approved by the institutional review board of Severance Hospital and Samsung Medical Center.

### *FGFR1* Fluorescence In Situ Hybridization

Fluorescence in situ hybridization (FISH) to detect *FGFR1* amplification was performed at the chromosomal level of the tissue microarray. We performed fluorescence signal detection with two probes on chromosome 8. The target probe is located on the *FGFR1* locus spanning 8p12 - 8p11.23 with spectrum orange (red) and CEN 8 with Spectrum Green (Abbott Molecular, Abbott Park, IL) following routine method. The evaluation was done independently by two experienced pathologists (Y.L.C. and H.K.) blinded to clinical information, and at least 100 nuclei per case were evaluated. *FGFR1* amplification was defined based on the previous studies.[25, 26] High *FGFR1* amplification was defined as if one of the following criteria is fulfilled: (1) *FGFR1*/CEN8 ratio is ≥ 2.0, (2) average number of *FGFR1* signal per nucleus ≥ 6.0, and (3) percentage of tumor cells containing ≥ 15 *FGFR1* signals or large clusters in ≥ 10% cells. Low *FGFR1* amplification was defined when the percentage of tumor cells containing ≥ 5 *FGFR1* signals is ≥ 50%.

### Mutation analysis

Genomic DNA was extracted from 388 formalin-fixed paraffin-embedded (FFPE) tissue specimens using an QIAamp DNA Micro kit (Qiagen, CA, USA) according to the manufacturer's instruction. The extracted DNA was used in a PCR amplification reaction. Based on the previous study in lung SqCC,[37] we evaluated the W290C, S320C, and K660E/K660N mutations in *FGFR2*, and R248C and S249C mutations in *FGFR3*. PCR amplification primers were designed to amplify following regions; W290C; 5′-TCCACAGTGGTCGGAGGAG-3′; 5′- AAAGTCCTCACCTTGAGAACCTTG-3′, S320C; 5′-CCTGGTTGGCCGTTATATTG-3′; 5′- TGTTTTGGCAGGACAGTGAG-3′, K660E/K660N; 5′- ATTCATCGAGATTTAGCAGCCAG-3′; 5′- ACATTCTGAGCCTCACCCC-3′, R248C and S249C; 5′-TGGCGGTGGTGGTGAG-3′; 5′- ATTCACCTCCACGTGCTTGA-3′.

PCR was carried out with the following conditions : initial denaturation at 95°C for 10 min, followed by 45 cycles for 30 s, 54°C for 60 s, 72°C for 45 s and final polymerization step of 72°C for 5 min in a GeneAmp PCR system 2720 (Life technologies, CA, USA). The amplified DNA product was visualized by gel electrophoresis and PCR products were sequenced using the Big dye terminator sequencing kit (Life technologies, CA, USA) according to the manufactures' instruction. Sequence reactions were the subjected to electrophoresis on an Applied Biosystems 3130XL DNA Analyzer (Life technologies, CA, USA).

### Statistical Analysis

We evaluated association between *FGFR1* amplification status and clinical significance using the χ^2^test or Fisher's exact test and trend was analyzed using linear regression. We also assessed whether *FGFR1* amplification influenced survival outcome using Kaplan-Meier curves with a log-rank test. Disease free survival (DFS) was defined from the time of surgery to initial relapse or death. Overall survival (OS) was measured from the time of surgery to death or last follow-up date, and 95% CIs were evaluated by survival analysis using the Kaplan-Meier method. Statistical significance was set at *P* < 0.05 for all analyses. Multivariate analysis was done using Cox regression analysis for following variables: sex, smoking status, stage, histology, adjuvant treatment, and FGFR1 status. All statistical analysis was performed using SPSS version 18.0 (SPSS, Chicago, IL).

### Disclosure of Potential Conflicts of Interest

The authors have no potential conflicts of interest to disclose.
